# P-2364. Estimated Additional Burden Averted for the 2022-2023 influenza season from Use of Cell-Based Influenza Vaccines Compared to Egg-Based Influenza Vaccines Among People 0-64 Years of Age in the United States

**DOI:** 10.1093/ofid/ofae631.2515

**Published:** 2025-01-29

**Authors:** Ian McGovern, Alicia N Stein, Mendel Haag

**Affiliations:** CSL Seqirus, Waltham, Massachusetts; CSL Seqirus, Waltham, Massachusetts; CSL Seqirus, Waltham, Massachusetts

## Abstract

**Background:**

Estimation of burden averted is useful to contextualize the impact of differentiated vaccines. Traditional influenza vaccine production in eggs can introduce egg-adaptive mutations. Cell-based influenza vaccines avoid egg-adaptation, potentially improving match to circulating influenza viruses and thereby vaccine effectiveness (VE). This study modeled the public health impact if all people aged 0-64 years vaccinated in the United States during the 2022-2023 influenza season had received either cell-based inactivated quadrivalent influenza vaccine (ccIIV4) or egg-based inactivated quadrivalent influenza vaccine (IIV4).

**Table 1:**
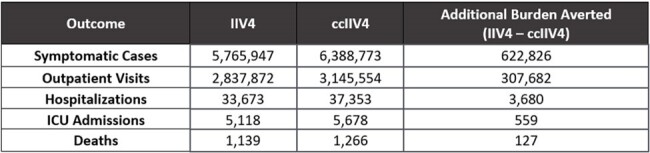
Number of outcomes prevented from use of IIV4 and ccIIV4, individuals 0—64 Years

**Methods:**

The modeling method used by the US Centers for Disease Control and Prevention (CDC) to estimate overall burden averted due to influenza vaccination was extended to a relative VE (rVE) context. The model utilized 2022-2023 CDC data on influenza vaccine uptake, influenza incidence, influenza-related healthcare resource (HRU) use and deaths. CDC estimates of absolute VE (aVE) (any vaccine) were used as the aVE of IIV4. We applied an rVE of 7.7% (0.9 to 13.9%), estimated in a 2022-2023 retrospective test-negative design study. Base-case results were validated with deterministic (DSA) and probabilistic (PSA) sensitivity analyses.

**Table 2:**
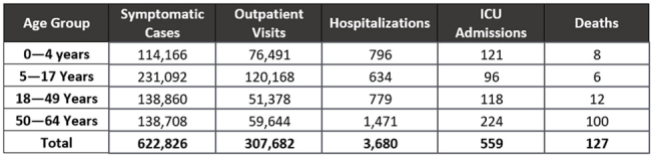
Number of additional outcomes prevented from use of ccIIV4 compared to IIV4, by age group

**Results:**

Table 1 shows the anticipated number of symptomatic influenza cases and associated HRU and deaths that would have been averted if all vaccinated people 0-64 years of age received either ccIIV4 or IIV4 and Table 2 shows the additional burden averted by age. Use of ccIIV4 would result in prevention of an additional 622,826 symptomatic illnesses, 307,682 outpatient visits, 3,680 hospitalizations, 559 intensive care unit (ICU) admissions, and 127 deaths compared to IIV4. DSA results showed that the rVE estimate was associated with the most variability, followed by influenza-related HRU and deaths. Mean PSA results were all within a 0.1% difference of base-case results.

**Conclusion:**

Use of ccIIV4 instead of IIV4 during the 2022-2023 influenza seasons in the US would have had a substantial public health impact on the population 0-64 years of age due to prevention of an additional 622,826 symptomatic illnesses as well as proportionate reductions in associated HRU and deaths.

**Disclosures:**

Ian McGovern, MPH, CSL Seqirus: Employee|CSL Seqirus: Stocks/Bonds (Public Company) Alicia N. Stein, MBiostats, PhD, CSL Seqirus: Employee|CSL Seqirus: Stocks/Bonds (Public Company) Mendel Haag, PhD, PharmD, CSL Seqirus: Employment|CSL Seqirus: Stocks/Bonds (Public Company)

